# Heavy metal accumulation potential in pomegranate fruits and leaves grown in roadside orchards

**DOI:** 10.7717/peerj.8990

**Published:** 2020-04-14

**Authors:** Şeyma Demirhan Aydın, Mine Pakyürek

**Affiliations:** Department of Horticulture, Faculty of Agriculture, Siirt University, Siirt, Turkey

**Keywords:** Heavy metal, Pollution, Roadside, Traffic, Fruit, Pomegranate, *Punica granatum* L.

## Abstract

This study was carried out to determine the possible heavy metal accumulation in fruits and leaves of Zivzik pomegranate (*Punica granatum* L.) grown in two different roadside orchards located in Pirinçli and Kapılı villages of Siirt province, Turkey. Leaf and fruit samples were collected from trees located at 0, 50, 100 m distances from the main roads. Plant samples were analyzed for cobalt (Co), nickel (Ni), cadmium (Cd), lead (Pb) and chromium (Cr) concentrations. The Co, Ni, Cd, Pb and Cr concentrations of fruit samples collected from Pirinçli village were ranged from 0.082 to 0.238 mg kg^−1^, from 1.160 to 1.559 mg kg^−1^, from 0.087 to 0.179 mg kg^−1^, 0.326 to 0.449 mg kg^−1^ and 0.606 to 1.054 mg kg^−1^, respectively. The Co, Ni, Cd, Pb and Cr concentrations of fruit samples from Kapılı village were between 0.085 and 0.137 mg kg^−1^, 1.042 and 1.123 mg kg^−1^, 0.037 and 0.076 mg kg^−1^, 0.277 and 0.520 mg kg^−1^ and 0.762 and 0.932 mg kg^−1^, respectively. Heavy metal concentrations of leaf samples from Pirinçli village varied from 0.191 to 0.227 mg Co kg^−1^, 2.201 to 3.547 mg Ni kg^−1^, 0.051 to 0.098 mg Cd kg^−1^, 0.535 to 0.749 mg Pb kg^−1^ and from 1.444 to 2.017 mg Cr kg^−1^. Similarly, the heavy metal concentration of leaf samples from Kapılı villages were between 0.213 and 0.217 mg Co kg^−1^, 2.160 and 2.511 mg Ni kg^−1^, 0.058 and 0.114 mg Cd kg^−1^, 0.579 and 0.676 mg Pb kg^−1^ and 1.688 and 1.518 mg Cr kg^−1^. The Co, Ni and Cr concentrations in fruit samples collected from 0, 50 and 100 meters to the main road in Pirinçli village were at statistically significant level, while only Ni concentration in leaf samples collected from 0, 50 and 100 meters to the main road was at significant level. In contrast, heavy metal concentrations in fruit and leaf samples collected from 0, 50 and 100 m to the main road in Kapılı village were not statistically significant level.

## Introduction

Due to the technological developments in the industrialization and urbanization process, the release of toxic pollutants as heavy metals into nature has become a major problem worldwide. Organic compounds such as heavy metals, pesticides, solvents and polyaromatic hydrocarbons are the most important environmental pollutants that can have toxic effects even at low doses (Memon & Schröder, 2009). Motor vehicles, mines, volcanic activities, energy production centers, fertilizers and pesticides used in agriculture and urbanization-industrial activities are among the sources of heavy metal accumulation. Heavy metals can be defined as metals with an atomic number of more than 40,04 and a density of more than 5 g/ cm^3^ and create toxicity and pollution. The most common heavy metal pollutants are Cd, Cr, Cu, Hg, Pb, Ni and Zn (Jadia & Fulekar, 2008).

The major cause of heavy metal pollution of air is motor vehicles (Seaword & Richardson, 1989). Vehicle traffic significantly affects agricultural lands located on the vicinity of highways by increasing the heavy metal pollution. The concentrations of Cd, Pb and Ni in agricultural lands increase due to the emissions of highway traffic (Hakerlerler et al., 1994). Due to the collapse of heavy metal pollutants released into the air by exhaust gases, heavy metal accumulation occurs both in the leaves and fruits of the plants and in the soil. Heavy metals cause the most important damages in plants in the biological cycle. Seed germination, seedling growth and development, regression in growth and development in plants, decrease in biomass production, flower and causes decrease in fruit formation, decrease in yield and deterioration in product quality. In addition, heavy metals disrupt photosynthetic activity, disrupt nitrogen cycle and binding, reduce chlorophyll content, cause deterioration in enzyme systems; It also has negative effects on intracellular mechanisms such as prevents the intake of other useful elements to plants.

Today, when environmentally friendly, sustainable production and life techniques are supported, the studies of many researchers trying to emphasize the negative effect of heavy metals in the environment attract attention. Hüseyinova et al. (2009) compared the heavy metal concentrations of *Corylus avellana, Alopecurus myosuroides, Helleborus orientalis, Glechoma hederacea, Calamintha nepeta* and *Urtica dioicaplanted* on polluted roadside fields with non-polluted fields in Ordu province. They reported statistically significant differences in heavy metal accumulation of plants located in “polluted” and “nonpolluted” areas.

In another investigation, *Platanus orientalis* L. trees which were located on two sides of a street with an average of 2100 vehicles passing per hour in Ankara were examined for the accumulation of Pb, Zn and Cu in the leaves, fruits and bark tissues for 5 months. In addition, the relationships between the source of the accumulation and the factors affecting the accumulation and monthly changes were also investigated. The accumulations of Pb, Zn and Cu were higher in the trees planted in the middle of the street and at the bus stops. The results revealed that the distribution of heavy metals in plant parts was different and the accumulation in leaves was higher than fruits and barks. Heavy metal accumulations increased with higher relative humidity and precipitation (Topa, 1995). Güneş & Çilalı (2018) searched leaf, fruit and soil samples taken from rosehip plants grown at 0, 25, 50, 100, 200, 500, 1,000 m distances to the highway. The collected samples were extracted according to the wet digestion method; cadmium, cobalt, chrome, copper, iron, manganese, nickel, lead and zinc heavy metals were read in the ICP device. As a result of the study, it has been concluded that rosehip is not affected by environmental pollution in relation to distance.

Concentrations of heavy metals in soils have significant effects on the concentrations of heavy metal uptake in plants. Therefore, increasing the levels of heavy metals in soils causes an increase in the concentrations of heavy metals in plants (Fergusson, 1990). Heavy metals are not uniformly distributed in plant tissues. Generally, the metal content of seeds or grains is lower compared to the vegetative organs. Therefore, the negative effect of heavy metals is less especially in cereals compared to the leafy forage and meadow plants. For example, the distribution of Cadmium (Cd) and Zinc (Zn) in different parts of maize grown in sludge treated soils has been reported as decreasing order of root-stem-leaf-crust-grain. The accumulation of heavy metals in the plants was reported as highest in root and lowest in fruit and grain. The distribution of heavy metals in various vegetative tissues is reported to be a function of xylem transport. Therefore, total concentration of an element in a specific tissue (except the stems) depends on the loss of water by transpiration and the duration of this process (Öztürk & Kiziroğlu, 1992).

By means of fertilizers used in agriculture, significant amounts of toxic elements are spread to the soil. Among these toxic elements are cadmium, lead, arsenic and copper. Phosphorus fertilizers and raw materials of these fertilizers play an important role in the contamination of these heavy metals into the soil (Köleli & Kantar, 2006). In another research, the effects of the emissions of Muğla-Yatağan Thermal Power Plant on heavy metal scopes of agricultural and forest soils around the power plant were investigated by Haktanır et al. (2010). For this purpose, 27 soil and 41 plant samples were collected from the distance between 721 m and 15 km away from the power plant, in other directions with the dominant wind direction. The Ni, Cd, Fe, Cu, Zn, Mn and S concentrations of soil samples have been determined. The results indicated that heavy metal and S concentrations of soils were not related to the distance to the power plant, but were mostly affected by the dominant wind direction. Gülser, Çığ & Sönmez (2011) studied to detect the phytoremediation capacities of ornamental plants used for landscape purposes. They were declared that the heavy metal contents of the roadside plants were higher in the study conducted on roadsides and away from the roads where traffic was dense.

The pomegranate grown in Şirvan district and surrounding villages of Siirt province in Southeast region of Turkey is called as Zivzik pomegranate which is highly preferred and consumed especially in the region. Zivzik pomegranate, which has a pleasant taste, aroma and fruit juice quality, is a coarse-grained fruit and suitable for long-term storage. Therefore, Zivzik pomegranate is also preferred outside of the region for fruit juice. It is well known that fruits and vegetables are an important part of human nutrition and provide vitamin and mineral support. Therefore, it is important to evaluate the risks to human health related to heavy metal accumulation especially in plants grown in urban areas and consumed as food (Schreck et al., 2012; Mombo et al., 2015; Shahid et al., 2015; Shahid et al., 2017). It is known that when heavy metals are high in the air, the intake of these heavy metals from the leaves and fruits causes significant health problems such as neurotoxic and carcinogenic diseases (Castro-Gonzalez & Mendez-Armenta, 2008; Jomova & Valko, 2010; Tokar, Benbrahim-Tallaa & Waalkes, 2011). Heavy metals pose danger and risk to all living organisms and humans. The exposed dose causes various diseases, especially cancer, depending on factors such as the person’s genetic structure and immune status, general health status, age, nutritional status. In addition to nutrition, it can cause respiratory and skin diseases (Çağlarırmak & Hepçimen, 2010).

We hypothesize that heavy metals may accumulate on pomegranate trees grown in roadside gardens and this accumulation can be high level in leaf samples than fruit. Because of this reason, we conducted our search to highlight the peril. This is the first study to determine the heavy metal contents of Zivzik pomegranate, which is an important source of income for local people in Siirt province. The aim of the study to determine the levels of cobalt, nickel, cadmium, lead and chromium elements, which are heavy metals found in exhaust gases formed as a result of burning fossil fuel and engine oils, in fruit and leaf samples collected from the Zivzik pomegranate orchards established on road sides and evaluate the effect of heavy metal pollution on pomegranate trees.

## Material & Methods

**Figure 1:**
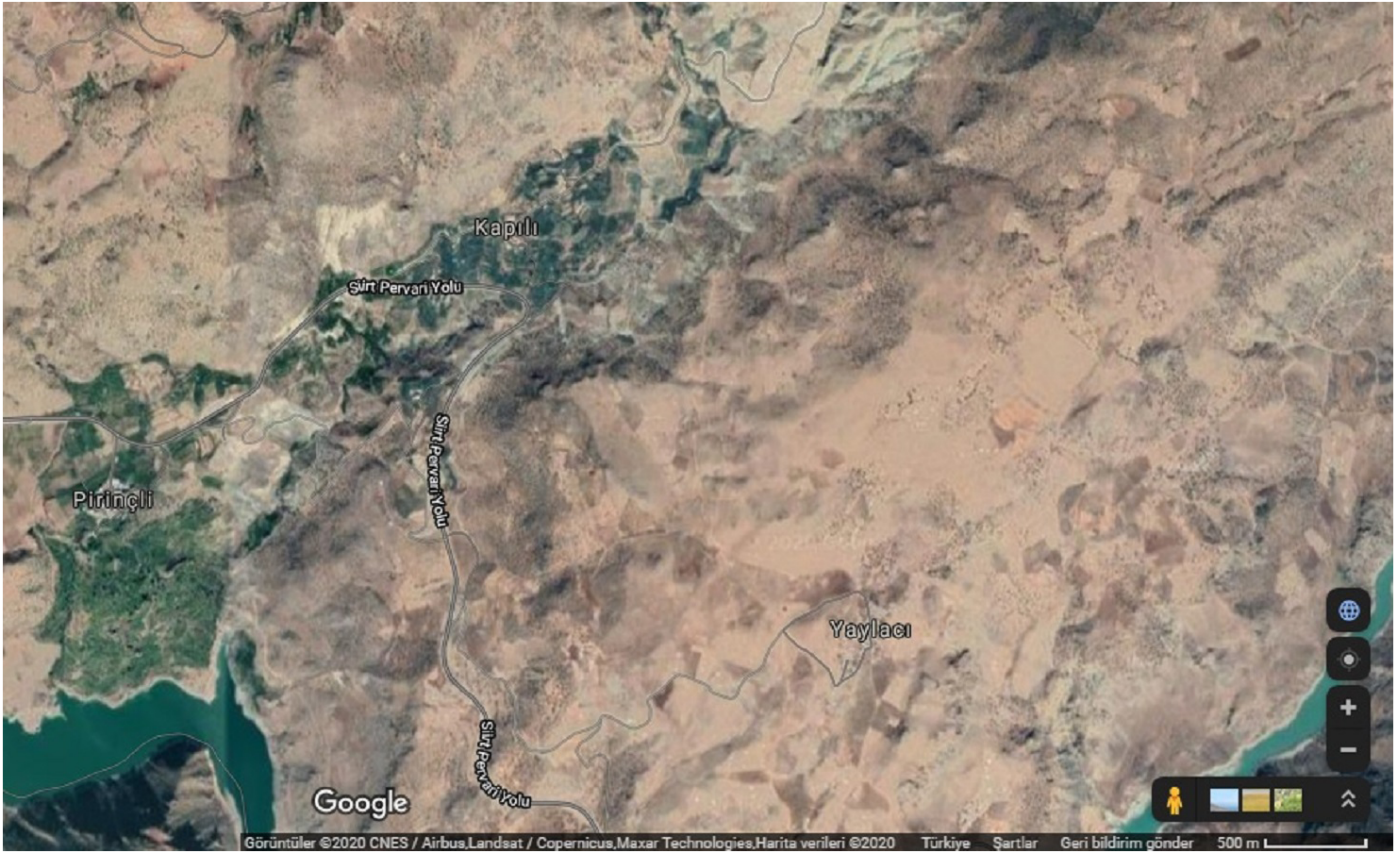
Satellige images of Kapılı and Pirinçli villages. Map credit: ©Google 2020 CNES/Airbus. Landsat/Copernicus. Maxar Technologies. Map data.

The orchards sampled were located in Pirinçli and Kapılı villages where Zivzik pomegranate is grown intensely in Siirt province of Southeast Anatolia region, Turkey (Fig. 1). Fruit and leaf samples were collected from two orchards located on the Siirt-Pervari Highway. According to the highways data, on average, 900 vehicles pass through this highway for daily. While the acceptable average carbon emission rate per gasoline vehicle passing through this road is 2.3 g km^−1^, for the diesel vehicle 0.9 g km^−1^ (Directorate-General of Highways of Turkey, 2019).

### Fruit and leaf samplings

Fruit and leaf samples of pomegranate trees located in rows at 0, 50 and 100 m distances from the main roadside were taken during the harvest at the end of the October. For each distance, starting from the first row parallel to the road, two trees from the beginning, two trees from the middle and two trees from the end of the row were selected and total of 18 trees were determined for each orchard. Four fruit samples and 12 leaf samples from each tree were collected.

### Heavy metal analysis

Leaf samples of Zivzik pomegranate were placed on hour glasses and dried at 78 °C for 48 h. Samples were then ground in a porcelain plate using a mallet, sieved through a 0.5 mm sieve and stored in vacuum bags. Fruit samples were frozen at −40 °C. The frozen samples were fractionated and granulated, and placed on the flasks and glass beakers. The samples were then taken into the lyophilizer to dry for 1 week. The dried pomegranate fruit samples were milled with a grinder and made ready for the chemical analysis.

The fruit and leaf samples were prepared for heavy metal analysis in a high-performance microwave assisted digestion system. In this system, 0.5 g of a sample, 7 ml of nitric acid (65%) and 1 ml of hydrogen peroxide (30%) were added into the Teflon wet combustion unit. Samples were digested at 200 °C and 45 bar pressure. After cooling, the solution was filtered using Whatman No. 42 filter paper and <0.45 µm Millipore filter paper. The solution was transferred into a volumetric flask with a final volume of 15 ml. The final volume was made up using distilled water (Milestone, 2008). The concentrations of Co, Cr, Ni, Cd and Pb were determined by an ICP-MS (Thermo Scientific ICAP Q model).

### Statistical analysis

The data were subjected to the one-way analysis (ANOVA) (Yıldız & Bircan, 2003). Mean values were statistically compared by the Least Significant Difference (LSD) test at P <0.05% significance level. Statistical analyses were performed using SPSS 20 statistical software (SPSS Inc., Chicago, IL, USA).

## Results

### Heavy metal concentrations of fruit samples

The results of ANOVA presenting the differences in Co, Ni, Cd, Pb and Cr concentrations of pomegranate fruits were given in Table 1. The distance to the road in Pirinçli village had a statistically significant effect on the Ni, Cr (*p* < 0.01) and Co (*p* < 0.05) concentrations of fruit samples while Cd and Pb concentrations did not significantly change with the distance. In contrast, the distance had no significant effect on heavy metal concentration of fruits collected from Kapılı village (Table 1).

**Table 1:** The results for variance analysis of fruit samples from the Pirinçli and Kapılı Villages.

			***PİRİNÇLİ***			***KAPILI***		
***Element***	**Source of variation**	**DF**	**MS**	**F**	**p**	**MS**	**F**	**p**
*Co*	Distance	2	0.019	8.78^*^	0.017	0.002	0.66	0.552
	Error	6	0.002			0.003		
*Ni*	Distance	2	0.125	11.53^**^	0.009	0.005	0.25	0.787
	Error	6	0.011			0.021		
*Cd*	Distance	2	0.007	0.53	0.614	0.001	1.10	0.393
	Error	6	0.014			0.001		
*Pb*	Distance	2	0.013	0.62	0.570	0.064	1.89	0.231
	Error	6	0.020			0.034		
*Cr*	Distance	2	0.152	19.84^**^	0.002	0.028	0.45	0.658
	Error	6	0.008			0.063		

**Notes.**

** Significant at *p* ≤ 0.01.

* Significant at *p* ≤ 0.05.

**Figure 2:**
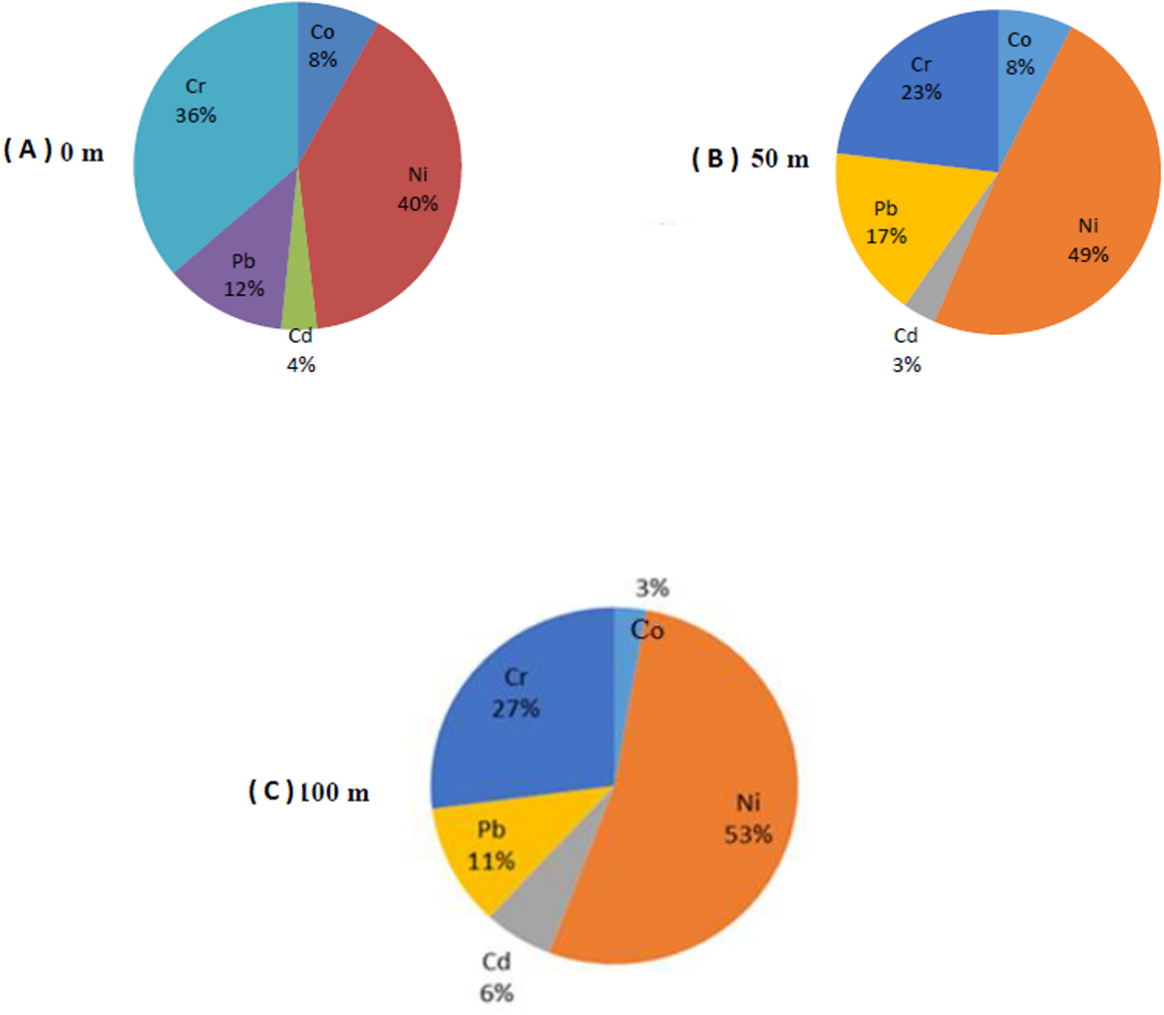
Heavy metal percentages of fruit samples collected at three different distances to the roadside. (A) Heavy metal percentages of fruit samples collected at 0 m distance to the roadside. (B) Heavy metal percentages of fruit samples collected at 50 m distance to the roadside. (C) Heavy metal percentages of fruit samples collected at 100 m distance to the roadside.

Descriptive statistics and LSD test results of heavy metal concentrations were given in Table 2. The mean Co concentrations of fruits in Pirinçli and Kapılı villages were 0.172 and 0.170 mg kg^−1^, respectively. The lowest mean Co concentration in both villages (0.082 and 0.085 mg kg^−1^) were obtained at 100 m distance, while the highest mean Co concentrations (0.238 and 0.137 mg kg^−1^) were obtained at 0 m distance (Table 2). The concentration of Co in fruits decreased with the increased distance from the roadside (**Fig. 2).

**Table 2:** Descriptive statistics of heavy metal concentrations (mg kg^−1^) in fruit samples by the distance.

**Distance to road**	Co*	Ni**	**Cd**	**Pb**	Cr**
	Pirinçli Village				
0 m	0.238 ± 0.079^#^ a ^&^	1.160 ± 0.068 b	0.102 ± 0.006	0.351 ± 0.019	1.054 ± 0.121 a
50 m	0.196 ± 0.021 a	1.285 ± 0.088 b	0.087 ± 0.008	0.449 ± 0.037	0.606 ± 0.031 c
100 m	0.082 ± 0.004 b	1.559 ± 0.142 a	0.179 ± 0.204	0.326 ± 0.244	0.796 ± 0.085 b
Mean	0.172 ± 0.081	1.334 ± 0.198	0.123 ± 0.111	0.375 ± 0.136	0.819 ± 0.209
	Kapılı Village				
0 m	0.137 ± 0.089	1.042 ± 0.163	0.068 ± 0.045	0.277 ± 0.192	0.762 ± 0.160
50 m	0.101 ± 0.033	1.094 ± 0.112	0.076 ± 0.030	0.520 ± 0.225	0.932 ± 0.398
100 m	0.085 ± 0.026	1.123 ± 0.151	0.037 ± 0.023	0.257 ± 0.121	0.765 ± 0.071
Mean	0.170 ± 0.054	0.094 ± 0.638	0.060 ± 0.034	0.351 ± 0.204	0.820 ± 0.233

**Notes.**

# Standard deviation values.

& The differences between the means in the same column are shown in separate letters (*p* ≤ 0.05).

According to the results obtained mean Ni concentrations of fruits in Pirinçli and Kapılı villages were 1.334 and 0.094 mg kg^−1^, respectively. The highest mean Ni concentrations in Pirinçli and Kapılı villages were 1.559 and 1.123 mg kg^−1^ (100 m), while the lowest values were 1.160 and 1.042 mg kg^−1^ (0 m) which was in the same statistical group with the 50 m distance (1.285 and 1.094 mg kg^−1^) (Table 2). The Cd concentrations in pomegranate fruits collected from Pirinçli and Kapılı villages ranged from 0.087 to 0.179 mg kg^−1^ and from 0.037 to 0.068 mg kg^−1^ while the mean Cd concentrations were 0.123 and 0.060 mg kg^−1^, respectively. The mean Pb concentrations of pomegranate fruits in Pirinçli and Kapılı villages were 0.375 and 0.351 mg kg^−1^, while Pb concentrations in fruit samples ranged from 0.326 to 0.449 mg kg^−1^ and 0.257 and 0.520 mg kg^−1^, respectively. The mean Cr concentrations in fruits collected from Pirinçli and Kapılı villages were 0.819 and 0.820 mg kg^−1^. The lowest Cr concentrations were 0.606 mg kg^−1^ in Pirinçli (50 m) and 0.762 mg kg^−1^ (0 m) in Kapılı while the highest Cr concentrations were 1.054 mg kg^−1^ (0 m) and 0.932 mg kg^−1^ (50 m) in Pirinçli and Kapılı villages.

The distribution of the heavy metal contents of the fruit samples collected from 0, 50 and 100 m distances in two different pomegranate orchards were shown in Fig. 2. The highest heavy metal content in pomegranate fruits collected from the orchards at 0 m distance was Ni with 40% (Fig. 2). Nickel was followed by Cr with 36%, Pb with 12%, Co with 8% and Cd with 4%. The results showed that Ni was the most common heavy metal accumulated in fruits due to the vehicle emissions. Similar to the 0 m distance, the highest heavy metal content at 50 m distance was Ni with 49%. Nickel was followed by Cr (23%), Pb (17%), Co (8%) and Cd (3%). Compared to the fruits taken from 0 m distance to the road, Ni and Pb concentrations increased and Cr and Cd concentrations decreased in 50 m distance. The Co content remained constant (Fig. 2). Similar to 0 and 50 m distances, the highest heavy metal content in fruits collected from trees at 100 m distance to the roadside was Ni with 53% (Fig. 2). The second highest heavy metal content in fruits was Cr (27%), followed by Pb (11%), Cd (6%) and Co (3%) (Fig. 2).

The relationship between the concentrations of Co, Ni, Cd, Pb and Cr of fruit samples collected from both villages was given in Table 3. A statistically significant (*r* = 0.656, *p* < 0.01) positive correlation was found between the Ni and Cd concentrations of pomegranate fruits (Table 3).

**Table 3:** Correlation matrix between heavy metal concentrations (mg kg^−1^) in fruit samples (*n* = 18).

	**Ni**	**Cd**	**Pb**	**Cr**
**Co**	−0.069	0.079	0.320	0.256
**Ni**		0.656^**^	0.023	0.059
**Cd**			−0.192	0.107
**Pb**				0.264

**Notes.**

** Significant at *p* ≤ 0.01.

### Heavy metal concentrations of leaf samples

The results of ANOVA testing the differences of Co, Ni, Cd, Pb and Cr concentrations of leaf samples according to the distance to the road were given in Table 4. The distance to the road in the Pirinçli village caused a statistically significant variation in the Ni concentration (*p* < 0.01) of the leaf samples (Table 4). However, the distance effect was not significant for the other heavy metals. Similarly, distance to road had no effect on all heavy metal concentrations studied in Kapılı village (Table 4).

**Table 4:** The results for variance analysis of leaf samples from Pirinçli and Kapılı villages.

			***Pirinçli Village***			***Kapılı Village***		
***Element***	**Source of Variation**	**DF**	**MS**	**F**	*p*	**MS**	**F**	*p*
*Co*	Distance	2	0.001	0.353	0.716	0.000	0.009	0.991
	Error	6	0.003			0.002		
*Ni*	Distance	2	1,443	28.092^**^	0.001	0.094	1.015	0.417
	Error	6	0.051			0.093		
*Cd*	Distance	2	0.002	1.140	0.381	0.002	1.278	0.345
	Error	6	0.002			0.002		
*Pb*	Distance	2	0.042	3.358	0.105	0.007	0.840	0.477
	Error	6	0.012			0.009		
*Cr*	Distance	2	0.247	0.869	0.466	0.001	0.021	0.979
	Error	6	0.285			0.031		

**Notes.**

** Significant at *p* ≤ 0.01.

Descriptive statistics of heavy metal concentrations in pomegranate leaf samples and LSD test results were given in Table 5.

**Table 5:** Descriptive statistics of heavy metal concentrations (mg kg^−1^) in leaf samples by the distance.

**Distance to road**	Co*	Ni**	**Cd**	**Pb**	Cr**
	Pirinçli Village				
0 m	0.227 ± 0.094^#^	2.201 ± 0.297 b	0.051 ± 0.011	0.535 ± 0.166	1.444 ± 0.376
50 m	0.191 ± 0.020	2.582 ± 0.099 b	0.098 ± 0.058	0.557 ± 0.066	1.763 ± 0.818
100 m	0.225 ± 0.032	3.547 ± 0.237 a	0.085 ± 0.032	0.749 ± 0.073	2.017 ± 0.207
Mean	0.215 ± 0.054	2.777 ± 0.632	0.078 ± 0.040	0.614 ± 0.140	1.742 ± 0.525
	Kapılı Village				
0 m	0.217 ± 0.034	2.160 ± 0.287	0.082 ± 0.051	0.579 ± 0.077	1.503 ± 0.109
50 m	0.217 ± 0.058	2.295 ± 0.407	0.058 ± 0.009	0.618 ± 0.064	1.518 ± 0.264
100 m	0.213 ± 0.024	2.511 ± 0.176	0.114 ± 0.053	0.676 ± 0.125	1.488 ± 0.107
Mean	0.216 ± 0.036	2.322 ± 0.305	0.085 ± 0.044	0.625 ± 0.090	1.503 ± 0.153

**Notes.**

# Standard deviation values.

With respect to the results the mean Co concentrations of leaves in the Pirinçli and Kapılı villages were 0.215 and 0.216 mg kg^−1^, seriatim (Table 5). The lowest mean Co concentration in Pirinçli village was 0.191 mg kg^−1^ at a distance of 50 m, while the highest mean Co concentration was 0.227 mg kg^−1^ at a distance of 0 m (Table 5). In Kapılı village, the lowest mean Co concentration was 0.213 mg kg^−1^ at a distance of 100 m, while the highest mean Co concentration was 0.217 mg kg^−1^ at the distances of 0 and 50 m (Table 5). The mean Ni concentrations of the leaves from Pirinçli and Kapılı villages were 2.777 and 2.322 mg kg^−1^, respectively. The highest mean Ni concentrations in Pirinçli and Kapılı villages were 3.547 and 2.511 mg kg^−1^ (100 m), while the lowest concentrations were 2.201 and 2.160 mg kg^−1^ (0 m) (Table 5). The mean Cd concentrations in the leaves were 0.078 and 0.085 mg kg^−1^ in Pirinçli and Kapılı villages, respectively. The mean values ranged from 0.051 to 0.098 mg kg^−1^ in Pirinçli village and from 0.058 to 0.114 mg kg^−1^ in Kapılı village (Table 5). The mean Pb concentrations of the leaf samples in Pirinçli and Kapılı villages were 0.614 and 0.625 mg kg^−1^, in order of. The mean Pb concentration in leaf samples of Pirinçli village was 0.535 mg kg^−1^ at 0 m distance, 0.557 mg kg^−1^ at 50 m distance and 0.749 mg kg^−1^ at 100 m distance. In Kapılı village, mean Pb concentration of leaf samples was 0.579 mg kg^−1^ at 0 m distance, 0.618 mg kg^−1^ at 50 m distance and 0.676 mg kg^−1^ at 100 m distance (Table 5). The average Cr concentrations of pomegranate leaves collected from Piriçli and Kapılı villages were 1.742 and 1.503 mg kg^−1^, respectively. The lowest mean value of Cr was 1.444 mg kg^−1^ at a distance of 0 m, while the highest mean value was 2.017 (100 m) mg kg^−1^ in Pirinçli village. In Kapılı village, the lowest average value for Cr was 0.488 mg kg^−1^ at 100 m distance, while the highest average value was 1.518 (50 m) mg kg^−1^.

**Figure 3:**
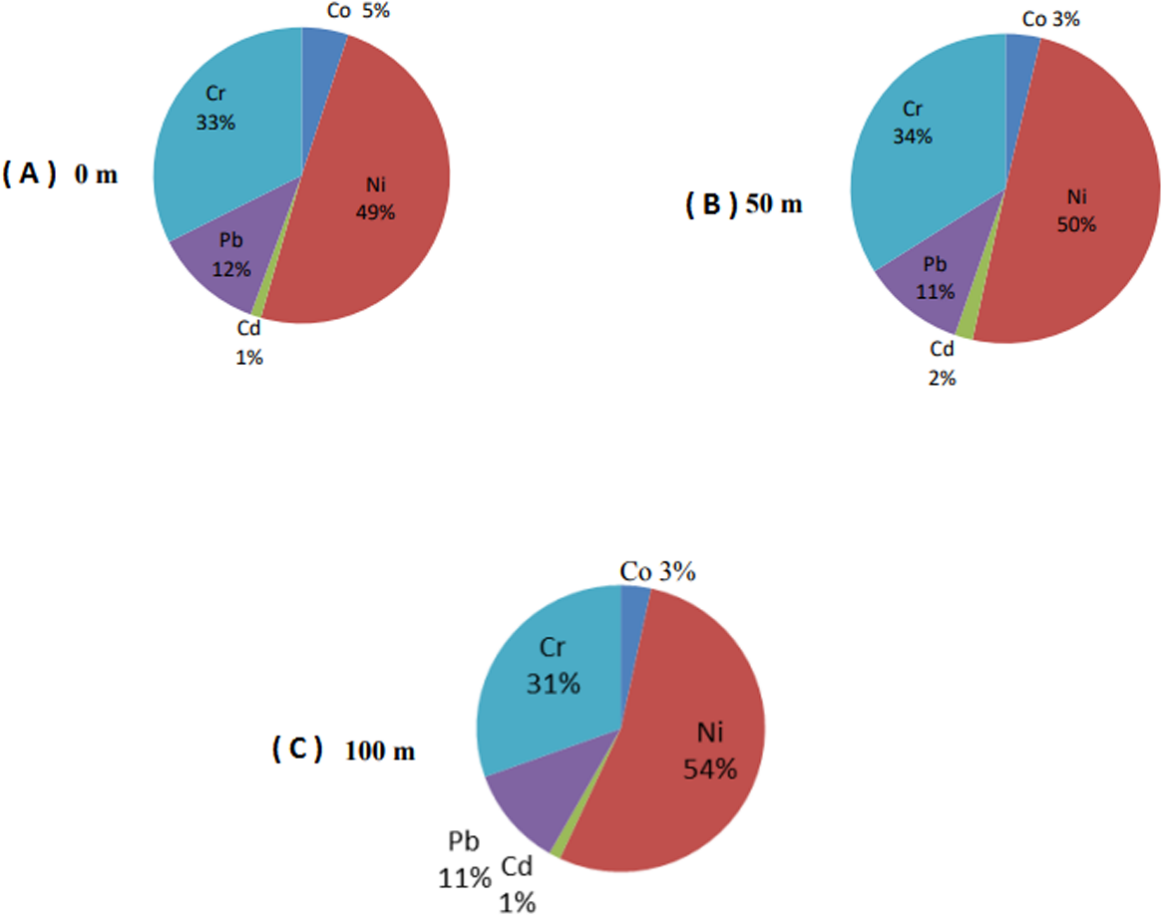
Heavy metal percentages of leaf samples collected from three varied distances to the roadside. (A) Heavy metal percentages of leaf samples collected at 0 m distance to the roadside. (B) Heavy metal percentages of leaf samples collected at 50 m distance to the roadside. (C) Heavy metals percentages of leaf samples collected at 100 m distance to the roadside.

The accumulation ratio of Ni (49%) in the leaf samples taken from the trees closest to the roadside (0 m) was higher than the other heavy metals. Nickel was followed by Cr (33%), Pb (12%), Co (5%) and Cd (1%) (Fig. 3). Although the ratios are different, the ranking of heavy metals in leaf samples was the same as the heavy metal contents in the fruits. Distribution of heavy metals in fruits and leaves depending on the distance to road were similar. The most common heavy metal found in pomegranate leaves taken from trees at 50 m distance to the roadside was Ni with 50%. Nickel was followed by Cr (34%), Pb (11%), Co (3%) and Cd (2%) (Fig. 3). The ranking of heavy metal distribution in 50 m distance was similar to that in 0 m distance. The distance to the road did not cause any difference in heavy metal accumulation in the leaves.

**Table 6:** Correlation matrix between heavy metal concentrations (mg kg^−1^) of leaf samples (*n* = 18).

	**Ni**	**Cd**	**Pb**	**Cr**
Co	0.267	0.188	0.259	−0.215
Ni		0.206	0.586^*^	0.541^*^
Cd			0.342	−0.194
Pb				0.413

**Notes.**

* Significant at *p* ≤ 0.05.

Similar to 0 and 50 m distances, Ni (54%) was the most common heavy metal found in pomegranate leaves taken from trees at 100 m distance from the roadside. Nickel was followed by Cr (31%), Pb (11%), Co (3%) and Cd (1%), respectively (Fig. 3). The distance from the road did not affect the order of Ni, Cr and Pb in the leaves. However, Co and Cd rankings in leaves taken from a distance of 100 m were different in comparison to fruits. The concentration of Co was higher than Cd in the leaf samples collected from the trees at 100 m distance while the opposite was observed in fruits.

The relationship between Co, Ni, Cd, Pb and Cr concentrations of pomegranate leaf samples collected from both villages was given in Table 6. Statistically significant (*p* < 0.05) positive correlations were obtained between Ni and Pb (*r* = 0.586) and Ni and Cr (*r* = 0.541) concentrations.

## Discussions

### Traffic effect on heavy metal concentration of fruits

Cobalt is an important heavy metal as well as an essential element in the structure of certain vitamins for animals and humans, essential for nitrogen fixation of legumes and also for similar activities of some bacteria and blue–green algae. The Co concentration of fruits reported in different studies has varied over a wide range. Gültaktı (2006) reported that the Co concentration in plants varied between 0.02 and 0.5 mg kg^−1^. The concentrations of Co in plants between 0.1 and 0.6 mg kg^−1^ have been stated as harmless to living organisms (Allen, 1989). The Co concentration of peach fruits collected from Selçuklu, Belevi, Davutlar and Kemalpaşa, Turgutlu and Salihli districts ranged from 3.2 to 9.6 mg kg^−1^ (Gönülsüz, 2000). The Co concentration of pear and quince grown in volcanic soils was found to vary between 0-1.32 and 0.36–1.20 mg kg^−1^ (Kaya, 2010). In another study conducted to determine heavy metal concentration of rosehip in the Eastern Anatolia region, the Co concentration was reported ranging from 0.033 and 0.083 mg kg^−1^ (Levent et al., 2009). The mean Co concentration in rosehip fruit samples collected from local herbal and grocery stores was 0.40 mg kg^−1^ (Başgel & Erdemoğlu, 2006). In our experiment the Co concentrations obtained were below the values in the other studies and the reported threshold values (Table 2). The Co concentration of fruit samples in Pirinçli village was not at toxic level. The decreased Co concentration with the increased distance to the road indicates that vehicle traffic is effective on the accumulation of Co in the pomegranate fruits.

Nickel is a heavy metal that emerges from burning fossil fuels and can be transferred from air to soil and living organisms. Nickel, which is necessary for plant nutrition, is generally low in agricultural lands and its concentration in agricultural soils up to 50 mg kg^−1^ was stated to be acceptable (Saatçi et al., 1988; Hakerlerler et al., 1994). Plants with low Ni content cannot benefit from nitrogenous fertilizers applied in the form of urea (Kaçar & Katkat, 2006). In present search the results revealed that vehicle traffic does not have a significant effect on Ni accumulation in pomegranate fruits in Pirinçli and Kapılı villages. Özbek et al. (1995) reported that critical toxic Ni level in sensitive plants is 10 mg kg^−1^ whereas the critical level in dry matter and moderate-sensitive plants is reported as 50 mg kg^−1^. According to the results of our search the Ni concentrations of pomegranate fruits collected from both villages were lower than the critical toxic level (Table 2). In another investigation, Kaya (2010) reported that Ni concentration in pear fruit grown in volcanic soils was between 5.46 and 16.56 mg kg^−1^, and in apple fruit between 0.22 and 5.48 mg kg^−1^, and in quince fruit between 9.08 and 11.64 mg kg^−1^. Morever, the Ni concentration of *Rubus fruticosus* fruit grown in an abandoned heavy metal mine site in Hungary was reported ranging from 4.041 to 4.657 mg kg^−1^ (Szentmihalyi et al., 2002). The Ni concentrations of Satsuma mandarin fruits in Gümüldür and Balçova districts, Izmir province were between 0.1 and 0.5 mg kg^−1^ in different research (Hakerlerler et al., 1994).

Cadmium, a mobile element in soil, can be easily up taken by plants and included in the food chain (Köleli & Kantar, 2006). Significant portion of Cd detected in plants and soils comes from precipitation of dust particles enriched with Cd. The Cd accumulation in roadside fields was between 0.2 and 1.0 mg m^−2^ in a year (Haktanır, 1987). The accumulations of Cd higher than 1.0 mg kg^−1^ in plant dry matter and 3.0 mg kg^−1^ in soil are reported causing toxic effects (Özbek et al., 1995). Therefore, in our experiment the Cd concentration in pomegranate fruits of Pirinçli village were lower than the critical toxic level reported. Industrial activities, phosphorus fertilizers, sewage wastes and atmospheric deposits play an important role in the spread of Cd on agricultural soils (Haktanır, 1987). In current study the Cd concentration in fruit samples did not linearly change depending on the distance to the road (Table 2). Because of this reason, vehicle traffic in studied orchards had no effect on Cd accumulation in the pomegranate fruits. On the other hand, Kaya (2010) reported that the Cd concentration in apple fruits grown in volcanic soils varied between 0.17 and 86.94 mg kg^−1^ and in pear fruit between 0.18 and 31.66 mg kg^−1^, and in quince fruit between 0.37 and 38.54 mg kg^−1^. Levent et al. (2009) found that Cd concentration in rosehip (*Rosa canina*) fruits in Eastern Anatolia region was between 0.0063 and 0.025 mg kg^−1^. In a similar study conducted in the Southeast Anatolia region, Şekeroğlu et al. (2008) indicated that the mean Cd content of *Rosa canina* was 33 mg kg^−1^. In another study, mean Cd concentration of *Rosa canina* collected from the local dealer in Turkey was reported as 0.07 mg kg^−1^ (Başgel & Erdemoğlu, 2006). Cadmium accumulation in organic cherry fruits in the Kemalpaşa district of İzmir province was reported to be between 0.03 and 0.12 mg kg^−1^ and in regular cherry orchards was between 0.06 and 0.20 mg kg^−1^ (Kulu, 2006).

Lead is released into the atmosphere as metal or compound and is toxic in both cases. Therefore, Pb can be classified as the most hazardous heavy metal that causes environmental pollution. Adverse effects of excess Pb accumulation on cell turgor and cell wall stability alter the plant water regime, leading to reduced leaf area and stoma movements. In addition, excess Pb reduces the anion and cation uptake of plants due to the decrease in root growth; thus, adversely affects plant nutrition (Sharma & Dubey, 2005). The accumulation of Pb in plant roots was reported higher compared to the shoots (Verma & Dubey, 2003), it was reported varying between 0.1 and 6.0 mg kg^−1^ in plants (Özbek et al., 1995). In present trial, the Pb concentration in pomegranate fruits in Pirinçli and Kapılı villages was lower than the reported critical threshold Pb concentration. No significant relationship was found between the Pb concentration of the fruit samples and the distance to the road. The difference in Pb concentrations of fruit samples collected from 3 sampling distances were not significantly different (Table 2), which shows that vehicle traffic does not have an impact on Pb accumulation on pomegranate fruits. A large part of Pb in nature results from the combustion of gasoline used as fuel in motor vehicles. The concentration of Pb that is not an essential nutrient for plants, in unpolluted soils ranges between 5 and 100 mg kg^−1^. The Pb concentration on the roadside soils in Los Angeles area where there is a heavy traffic was reported reaching to 2400 mg kg ^−1^(Kojo & Lodenius, 1989; Haktanır, 1987). Dürüst et al. (2004) reported that Cd concentration below 150 mg kg^−1^ does not poses any threat on human and plant health, however; Pb concentration exceeding 300 mg kg^−1^, is risky for human health. In another search, the Pb concentration in rosehip (*Rosa canina*) fruits at Eastern Anatolia region ranged from 0.111 to 0.273 mg kg^−1^ (Levent et al., 2009). In a similar study with rosehip, Güneş & Çilalı (2018) reported that Pb concentration in leaf and fruit samples of rosehip plants grown on Amasya-Tokat highway was below the threshold value. The concentration of Pb varied between 0.22 and 5.48 mg kg^−1^ in apple fruits, in pear fruits between 0 and 3.38 mg kg ^−1^ andin quince fruits between 0.0 and 2.82 mg kg^−1^ grown in volcanic soils (Kaya, 2010).

The initial impacts of chromium are observed on seed germination when the Cr concentration reaches toxic level in the plant. The Cr, which is usually less than 0.1 µg m^−3^ in air and less than 1 µg l^−1^ in uncontaminated water, can be found everywhere in nature. In general, Cr in soils ranges between 2 and 60 mg kg^−1^ (Özbek et al., 1995). Available Cr concentration in Uludağ University agricultural research field was reported between 0.02 and 0.60 mg kg^−1^ (average 0.25 mg kg^−1^) (Deveciler, 2005).

In this research, the accumulation of Cr was higher in the fruit samples taken from the trees closer to the roadside compared to other distances. Therefore, the traffic is considered effective in the increase of Cr accumulation of the pomegranate fruits. Similar to the Pb, significant portion of Cr taken up by the plant is given out by the root or root surface, while only a small portion is transferred to the upper parts of the plant. The Cr ratio in plant parts decreases from root to grain and fruit, respectively (Gültaktı, 2006). High Cr concentration prevents stem cell division and elongation and adversely affects root growth which reduces the absorption of nutrients and water from the soil and consequently reduces plant growth (Jain, Srivastava & Madan, 2000; Özbek et al., 1995). The Cr in soil ranges between 5 and 100 mg kg^−1^. The concentration over 100 mg Cr kg^−1^ in plant dry matter creates a toxic effect for many plants (Özbek et al., 1995). Fruit Cr concentrations determined in this study for both (Pirinçli and Kapılı) villages were lower than the toxic threshold levels reported. Kaya (2010) found the similar results with our study that the Cr concentration in the quince, grape and walnut fruit samples between 13.16–20.32 mg kg^−1^, 13.76–27.60 mg kg^−1^ and 20.13–34.60 mg kg^−1^, respectively.

### Traffic effect on heavy metal concentration of leaves

The vehicle traffic had no effect on Cr accumulation in the leaves of pomegranate plants in Pirinçli and Kapılı villages. The Co concentration of leaf samples did not linearly change with the distance to the road. The Cr concentrations pomegranate leaves were generally below the concentrations and threshold values reported in other studies. The Co concentration of peach leaf samples in Selçuklu-Belevi-Davutlar and Kemalpaşa-Turgutlu-Salihli districts in Aegean region was reported between 4.8 and 9.6 mg kg^−1^ (Gönülsüz, 2000). Similar to the findings in this study, Güneş & Çilalı (2018) reported that Co concentration was at insignificant level in the leaves of rosehip plants that grown naturally around Amasya-Tokat highway.

The results indicated that vehicle traffic does not have an impact on Ni accumulation in pomegranate leaves. The Ni concentration of leaves were below the findings and threshold values reported in previous studies. Hakerlerler et al. (1994) reported that leaf Ni concentrations in satsuma mandarin orchards located in Gümüldür and Balçova districts of Izmir province was between 2 and 5 mg kg^−1^. Despite the high Ni concentration of soils in the study area, low Ni concentrations in leaves and fruits were attributed to the relationship between Ni uptake and soil pH, clay and organic matter contents and plant species. Güneş & Çilalı (2018) investigated the heavy metal pollution of soils related to the distance to the roadside and observed that Ni concentration in the leaves did not increase in parallel to the increase in distance to the road.

The traffic in study area had no significant impact on Cd increase in pomegranate leaves. Leaf Cd concentrations were below the concentrations and limit values reported in other studies. Kulu (2006) reported that Cd concentration of leaves in organic cherry orchard was between 0.06 and 0.32 mg kg^−1^ and in regular cherry orchard was between 0.09 and 0.21 mg kg^−1^. The Cd concentration of peach leaves in the Selçuk-Belevi-Davutlar region was reported between 0.20 and 0.40 mg kg^−1^ (Gönülsüz, 2000). In this study, a linear change in leaf Cd concentration was not detected depending on the distance to the road.

The concentration of Pb in leaves increased as the distance to the road increased. The traffic on roads of both villages had no significant effect on Pb accumulation in leaves of pomegranate plants. The Pb concentrations of pomegranate leaves were below the values reported in the other studies and the threshold values. Lead concentration of peach leaves in Selçuk, Belevi and Davutlar regions was determined between 6.0 and 10.1 mg kg^−1^ which indicated excess Pb accumulation (Gönülsüz, 2000). Similar to the findings of the current study, Güneş & Çilalı (2018) found that the leaves and fruits of the rosehip plants around the Tokat-Amasya highway were not affected by Pb pollution in the soil or in the surrounding area.

The Cr concentrations of leaf samples obtained in this study were below the concentrations and reported limit values in other studies. The toxicity limit value of Cr in plants was reported as 1–2 mg kg^−1^ (Sauerbeck, 1982) and Scheffer & Schachtschabel (1989) reported that Cr concentration in plant leaf samples ranged between 0.1 and 1.0 mg kg^−1^. The Cr concentrations of leaves collected from organic cherry orchards were between 0.002 and 0.20 mg kg^−1^, while those collected from regular cherry orchards were between 0.002 and 0.80 mg kg^−1^ (Kulu, 2006). Gönülsüz (2000) indicated that the Cr concentration of peach leaves in Selçuk-Belevi-Davutlar region was between 0.50 and 3.03 mg kg^−1^ and that concentration increased as the distance to the road increased.

The results showed that the ratio of Ni element in pomegranate fruit and leaf samples increased as moved away from the roadside. The increase in Ni content of fruits can be explained by the effect of wind on Ni transport. In addition, the essential metals such as Fe, Mn, Zn, Cu, Mo, and Ni for the plants are taken up and accumulated by plants (Williams, Pittman & Hall, 2000). Since Ni is a vital element for plants, it has been determined that it is found in a higher rate in fruit samples taken from lines far away from the roadside. Because of this reason Ni accumulation in plants can increase as moved away from the roadside.

The results specifically confirmed our hypothesis that the accumulation of heavy metals in leaf samples are higher than the values of fruit samples. The results also indicated mobility of Co, Cr, and Ni is lower than that of Pb. In addition, the distance from contaminant source, here vehicle exhaust, significantly influences the availability and uptake of these metals. By contrast, the Pb is more mobile and causes greater concern for harvestable fruits grown in close proximity to roads. Some metals are subject to bioaccumulation because they are not mobile. Others are mobile and have potential of transferring either through soil profile down to ground water or through plant-root uptake (Businelli et al., 2009a; Businelli, Massaccesi & Onofri, 2009b; Kouame et al., 2010). Metal mobility is impressed by soil properties such as organic matter, oxides as well as soil structure and profile development. In long term models of metal mobility, type of soil, vegetation, hydrology, use of land and biological activity play a key role (De Matos et al., 2001; Businelli, Massaccesi & Onofri, 2009b; Lal, 2010; Kouame et al., 2010; Ashraf, Maah & Yusoff, 2011; Mehes-Smith et al., 2013). In plants heavy metal accumulation is probably related with their physiological and anatomical features. Heavy metal accumulation on leaf surfaces takes place through stomata, cuticular cracks, lenticels, ectodesmata, and aqueous pores. Heavy metal accumulation changes according to the structure of plant canopy, leaf inclination angle, branch density, leaf lamina morphologic anatomical structure, and leaf area (Shahid et al., 2017) Moreover, heavy metal accumulation hinges on plant species, genetic structure and plant age. Results of an experiment conducted by (Turkyilmaz et al., 2018) also supported the differences in heavy metal accumulation based on both plant species and traffic density. In traffic-dense areas, they measured the greatest concentrations of Cu, Ni, and Fe in *Prunus cerasifera*; Ca, Mg, and Mn in *Ailanthus altissima*; Cr and Zn in *Elaeagnus angustifolia*; and Pb and Cd in *Tilia tomentosa*.

## Conclusions

The concentrations of Ni, Cr, Pb, Cr and Cd were determined in fruits and leaves of pomegranate trees growing on rows at 0, 50 and 100 m distances to the Siirt-Pervari Highway in Siirt province, Turkey. The results are important to understand the influence of heavy metals on pomegranate trees, to determine the capacity of plants accumulating the heavy metals studied, and in this context to evaluate the impact of heavy metal accumulation on human health.

The differences in the locations of the villages, wind direction, soil characteristics, location of roads and vehicle traffic density have been expected to cause the differences in the heavy metal accumulation in leaves and fruits. The difference between the mean Co, Ni and Cr concentrations in Pirinçli village was statistically significant. The Co and Cr concentrations had the highest mean values in fruits collected from 0 m distance, while the Ni in fruits was at the highest concentration at 100 m. In the Kapılı village, the difference between the mean values of heavy metal concentrations in the fruits collected from all three distances was statistically insignificant. The difference between the mean heavy metal contents in the leaves collected from 0, 50 and 100 m distances in Pirinçli village was only significant for Ni concentration. The highest Ni concentration in leaves was obtained at the 100 m distance. The difference between the mean heavy metal concentrations of the leaves in Kapılı village was not statistically significant. The results concluded that Co, Ni and Cr concentrations of the pomegranate fruits in Pirinçli village and only Ni concentration of the leaves were found to be statistically significant.

The concentration of Ni in fruits was higher than other four heavy metals in both villages. Nickel was followed by Cr, Pb, Cd and Co in 0 to 50 m distances, while the ranking in 100 m was Ni, Cr, Pb, Co and Cd, respectively. The heavy metal content in the pomegranate leaves was in the order of Ni, Cr, Pb, Co and Cd. The heavy metal contents of fruit and leaf samples collected from 0 and 50 m distances were similar. The results of the study indicated that the fruits and leaves of the pomegranate trees grown in Pirinçli village had a higher heavy metal accumulation compared to the fruit and leaf samples in the Kapılı village. In both villages, the most common heavy metals of fruits and leaves were Ni, Cr and Pb, respectively. The vehicle traffic mostly caused accumulation of these heavy metals.

The results obtained and the observations during field studies indicated that the producers are not very careful in determining the location of the pomegranate orchards or they do not know how the vehicle pollution may affect the quality of fruits in the orchards established on the main roads. Pomegranate trees should be adequately fertilized by using required plant nutrients and the levels of elements that may have toxic effects should also be determined. Heavy metal accumulation threatens human health as well as plants and all living organisms that feed by plants. The results revealed that vehicle traffic may increase the heavy metal accumulation in pomegranate plants on roadside orchards. In order to prevent the negative consequences of heavy metal accumulation on fruits, special care should be taken to establish the new gardens within one to two km away from the roadside. The results indicated that necessary legal and administrative arrangements are needed to enforce the establishment of orchards away from the roadsides. Necessary trainings should be provided for producers to act in compliance with the new regulations and raise public awareness on healthy food consumption.

##  Supplemental Information

10.7717/peerj.8990/supp-1 Supplemental Information 1 Dataset of analysis of heavy metals

## Additional Information and Declarations

### Data Availability

The following information was supplied regarding data availability: The raw data is available as a Supplemental File.
